# Supplementary material for: Discovery of essential kinetoplastid-insect adhesion proteins and their function in Leishmania-sand fly interactions
Source: Nat Commun. 2024 Aug 13;15:6960. doi: 10.1038/s41467-024-51291-z (PMC11322530; doi:10.1038/s41467-024-51291-z)

Supplementary Information

Discovery of essential kinetoplastid-insect adhesion proteins and their function in *Leishmania*-sand fly interactions

Ryuji Yanase[1,7,8*], Katerina Pruzinova[2], Barrack O. Owino[1], Edward Rea[1], Flávia Moreira-Leite[1,9], Atsushi Taniguchi[3,4], Shigenori Nonaka[3,5,6], Jovana Sádlová[2], Barbora Vojtkova[2], Petr Volf[2*], Jack D. Sunter[1*]

[1]Department of Biological and Medical Sciences, Oxford Brookes University, Oxford, United Kingdom. [2]Department of Parasitology, Faculty of Science, Charles University, Prague, Czech Republic. [3]Laboratory for Spatiotemporal Regulations, National Institute for Basic Biology, Okazaki, Japan. [4]Research Center of Mathematics for Social Creativity, Research Institute for Electronic Science, Hokkaido University, Sapporo, Japan. [5]Spatiotemporal Regulations Group, Exploratory Research Center for Life and Living Systems, Okazaki, Japan. Department of Basic Biology, School of Life Science, SOKENDAI, Okazaki, Japan. [7]School of Life Sciences, University of Nottingham, Nottingham, United Kingdom. [8]Department of Genetics and Genome Biology, University of Leicester, Leicester, United Kingdom. [9]Central Oxford Structural Molecular Imaging Centre (COSMIC), Department of Biochemistry, University of Oxford, Oxford, United Kingdom.

***For correspondence**: ryuji.yanase@nottingham.ac.uk, volf@cesnet.cz, jsunter@brookes.ac.uk

Supplementary Figures

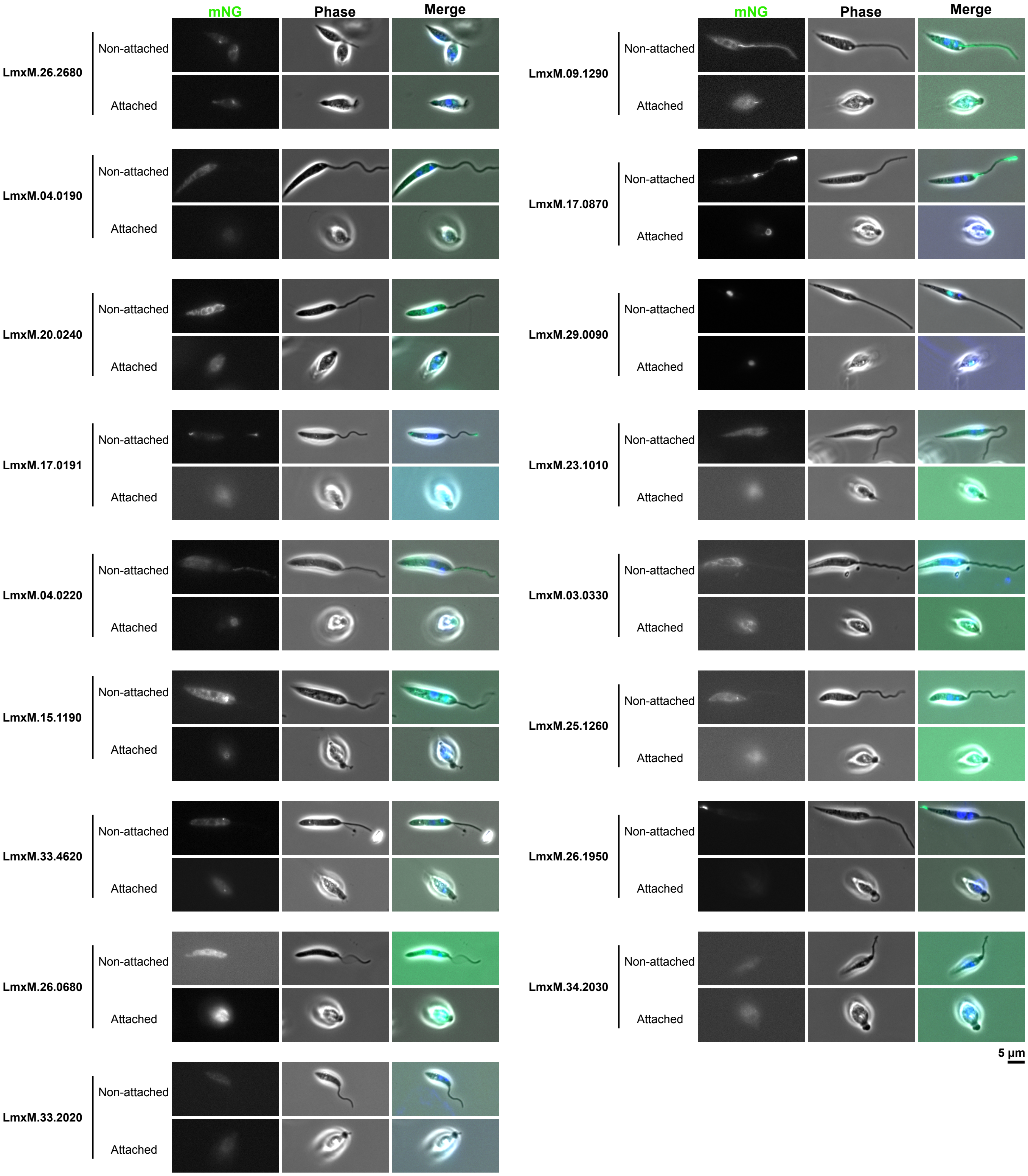

Supplementary Fig. 1. mNeonGreen tagging screening of the identified proteins in the comparative proteomics analysis. Localisation of the identified proteins in the comparative proteomics which are endogenously tagged with mNG in non-attached *in vitro* promastigotes and attached *in vitro* haptomonad-like promastigotes are shown. Representative images from n = 1 sample preparation are shown. In the merged images, the overlays of the phase contrast, mNG (green) and Hoechst-stained DNA (blue) images are shown.

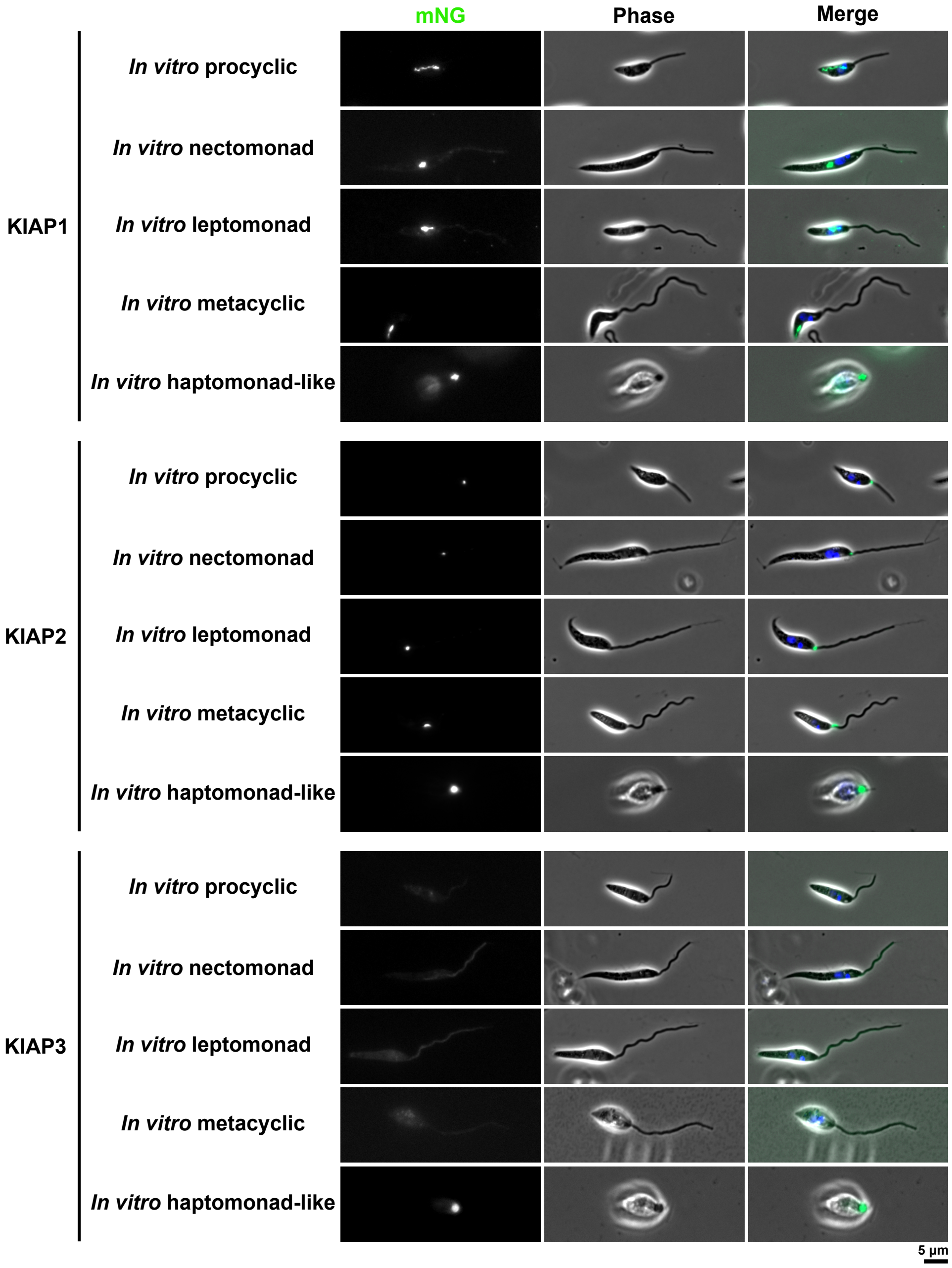

Supplementary Fig. 2. Localisation of KIAPs in the different promastigote stages. Localisation of mNG-tagged KIAP1-3 in *in vitro* promastigotes. 100 random *in vitro* promastigotes were classified into the following five different promastigote stages based on their morphology, and the number of cells in each promastigote stage was counted; (i) *in vitro* procyclic promastigote: KIAP1 (44/100 cells), KIAP2 (33/100 cells), KIAP3 (28/100 cells), (ii) *in vitro* nectomonad promastigote: KIAP1 (19/100 cells), KIAP2 (36/100 cells), KIAP3 (17/100 cells), (iii) *in vitro* leptomonad promastigote: KIAP1 (32/100 cells), KIAP2 (28/100 cells), KIAP3 (52/100 cells), (iv) *in vitro* metacyclic promastigote: KIAP1 (5/100 cells), KIAP2 (3/100 cells), KIAP3 (3/100 cells), and (v) *in vitro* haptomonad-like promastigote: KIAP1 (0/100 cells), KIAP2 (0/100 cells), KIAP3 (0/100 cells). Representative images from n = 1 sample preparation are shown. The images of the haptomonad-like promastigotes were taken from the images captured in the KIAPs localisation assay in Fig. 1b. In the merged images, the overlays of the phase contrast, mNG (green) and Hoechst-stained DNA (blue) images are shown. Source data are provided as a Source Data file.

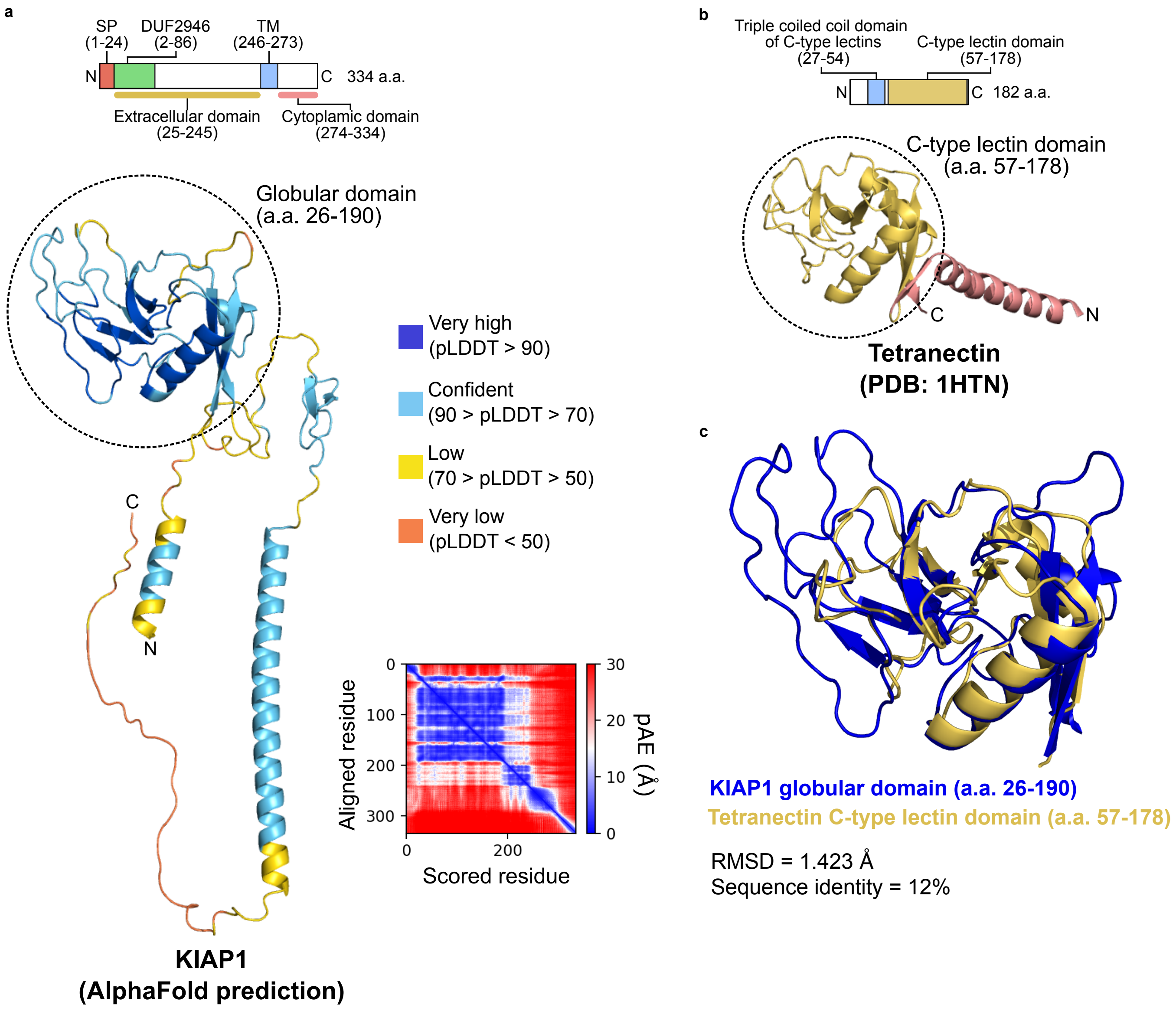

Supplementary Fig. 3. AlphaFold prediction shows a globular domain of KIAP1 has a similar 3D structure with that of a C-type lectin domain. a, Domain structure and 3D structure of KIAP1 predicted by AlphaFold. Globular domain of KIAP1 is indicated by a dotted circle (amino acids (a.a.) 26-190). Colours of the 3D structure represent the predicted local distance difference test score (pLDDT) of the AlphaFold prediction. The corresponding plot of predicted aligned error (pAE) of the residues is shown at bottom right. **b**, Domain structure and 3D structure of human Tetranectin (PDB: 1HTN)[1]. C-type lectin domain is shown in yellow in the domain and 3D structure. **c**, Superimposition of the 3D structures of the KIAP1 globular domain (blue; a.a. 26–190) and Tetranectin C-type lectin domain (yellow; a.a. 57–178). The root-mean-square deviation (RMSD) between two protein structures calculated using PyMOL was 1.423 Å and the sequence identity was 12%.

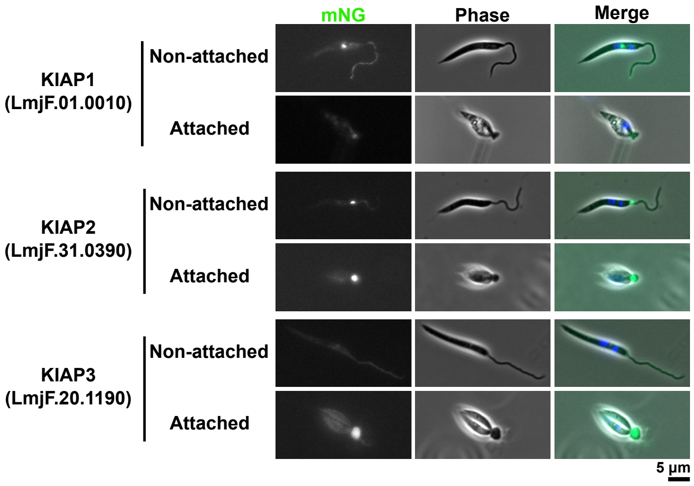

Supplementary Fig. 4. Localisation of mNeonGreen-tagged KIAPs in *Leishmania major* non-attached *in vitro* promastigote and *in vitro* haptomonad-like promastigotes. KIAP1-3 respectively were C-terminally tagged with mNG in *Leishmania major* cells. Localisation of KIAPs in non-attached *in vitro* promastigotes and *in vitro* haptomonad-like promastigotes attached for 24 h on a glass coverslip were confirmed with fluorescence microscopy. Representative images from at least n = 2 independent sample preparations are shown. In the merged images, the overlays of the phase contrast, mNG (green) and Hoechst-stained DNA (blue) images are shown.

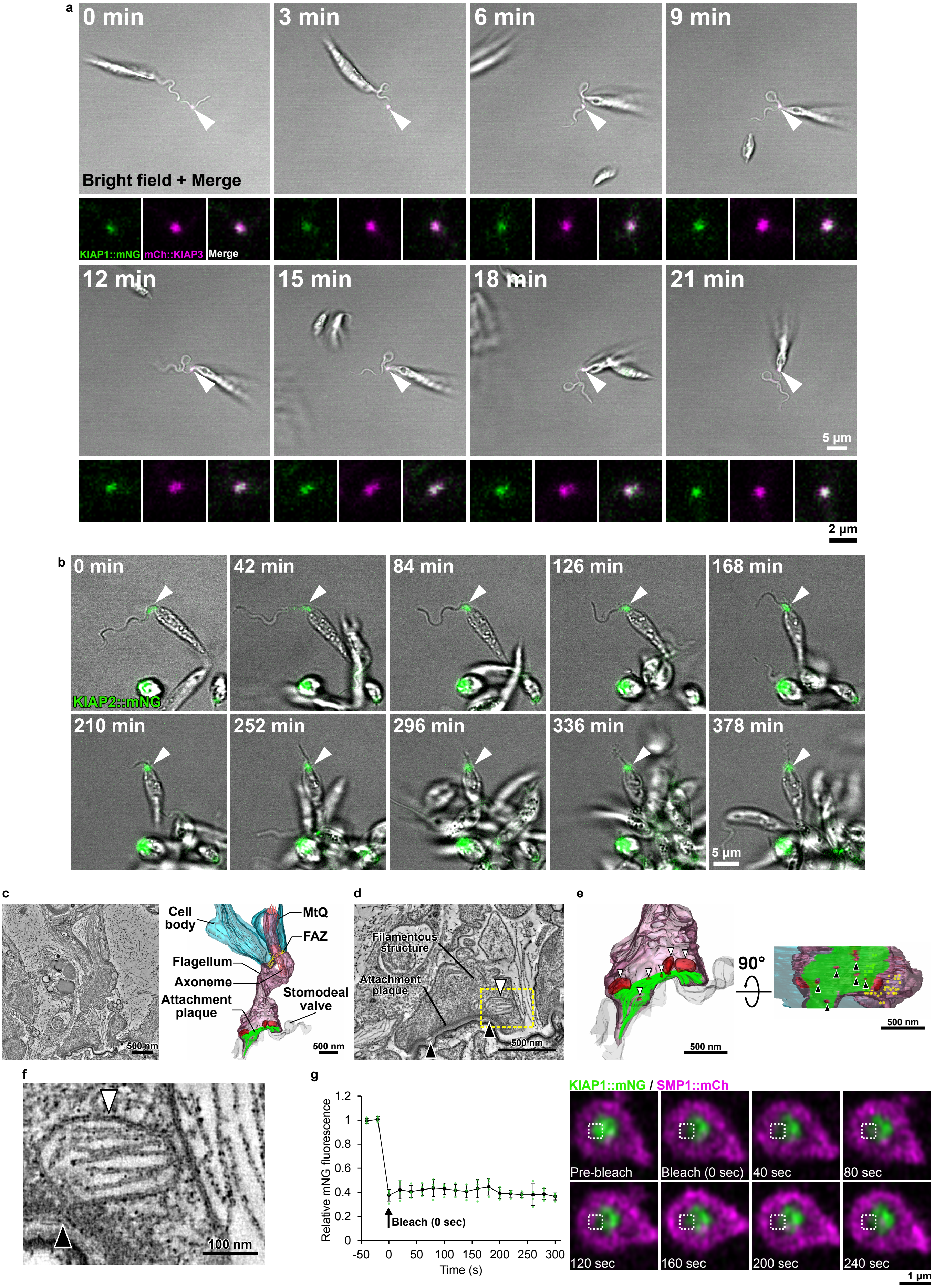
a
0 min
3 min
6 min
9 min
Bright field + Merge
KIAP1::mNG
mCh::KIAP3
Merge
12 min
15 min
18 min
21 min
5 µm
2 µm
b
0 min
42 min
84 min
126 min
168 min
KIAP2::mNG
210 min
252 min
296 min
336 min
378 min
5 µm
c
500 nm
Cell body
MtQ
FAZ
Flagellum
Axoneme
Attachment plaque
Stomodeal valve
500 nm
d
Filamentous structure
Attachment plaque
500 nm
e
90°
500 nm
500 nm
f
100 nm
g
Relative mNG fluorescence
Bleach (0 sec)
Time (s)
KIAP1::mNG / SMP1::mCh
Pre-bleach
Bleach (0 sec)
40 sec
80 sec
120 sec
160 sec
200 sec
240 sec
1 µm

Supplementary Fig. 5. Time-lapse observation of the development of KIAP1, 2 and 3 during adhesion processes of *in vitro* haptomonad-like promastigotes. a, Sequential frames (at 3 min intervals) from a time-lapse movie of adhesion of an *in vitro* haptomonad-like promastigote and development of KIAP1::mNG and mCh::KIAP3. Representative images from n = 2 independent sample preparations are shown. Merged images of bright field and mNG and mCh signals are presented, with adhesion starting point (arrowheads) remaining fixed. Confocal images of KIAP1::mNG (green) and mCh::KIAP3 (magenta) at each time point, along with their merged images, are shown below. **b**, Sequential frames (at ~40 min intervals) from a time-lapse movie of a complete adhesion process of an *in vitro* haptomonad-like promastigote and development of KIAP2::mNG. Representative movie stills from n = 2 independent sample preparations are shown. Adhesion starting point (arrowheads) remain fixed. **c**, Serial tomogram slice and 3D reconstruction of an *in vivo* haptomonad cell in the sand fly. Representative tomogram slice from n = 5 different cells is shown. Vesicles near the attachment plaque (green) are shown in red. **d**, Magnified view of the tomogram showing that multiple vesicles (white arrowheads) are seen near the attachment plaque and the plaque is interrupted at the fusion of the vesicle and flagellar membrane (black arrowheads). **e**, Magnified view of the 3D reconstruction showing that multiple vesicles (white arrowheads) are seen near the attachment plaque (green) and the plaque is interrupted at the fusion of the vesicle and flagellar membrane (black arrowheads). **f**, Magnified view of the vesicle near the attachment plaque (yellow dotted box in d) revealing structures inside the vesicle resemble the filamentous extracellular matrix. **g**, Long-time FRAP experiment of KIAP1::mNG. The relative fluorescence intensity (the average fluorescence intensity before photobleaching as 1) changes before and after photobleaching is shown, with sequential frames (at 40 sec intervals) of KIAP1::mNG and SMP1::mCh. Data represent mean ± SD (n = 4 independent experiments). Values from each experiment are shown with green dots. Dotted white boxes indicates the area of photobleaching. Source data are provided as a Source Data file.

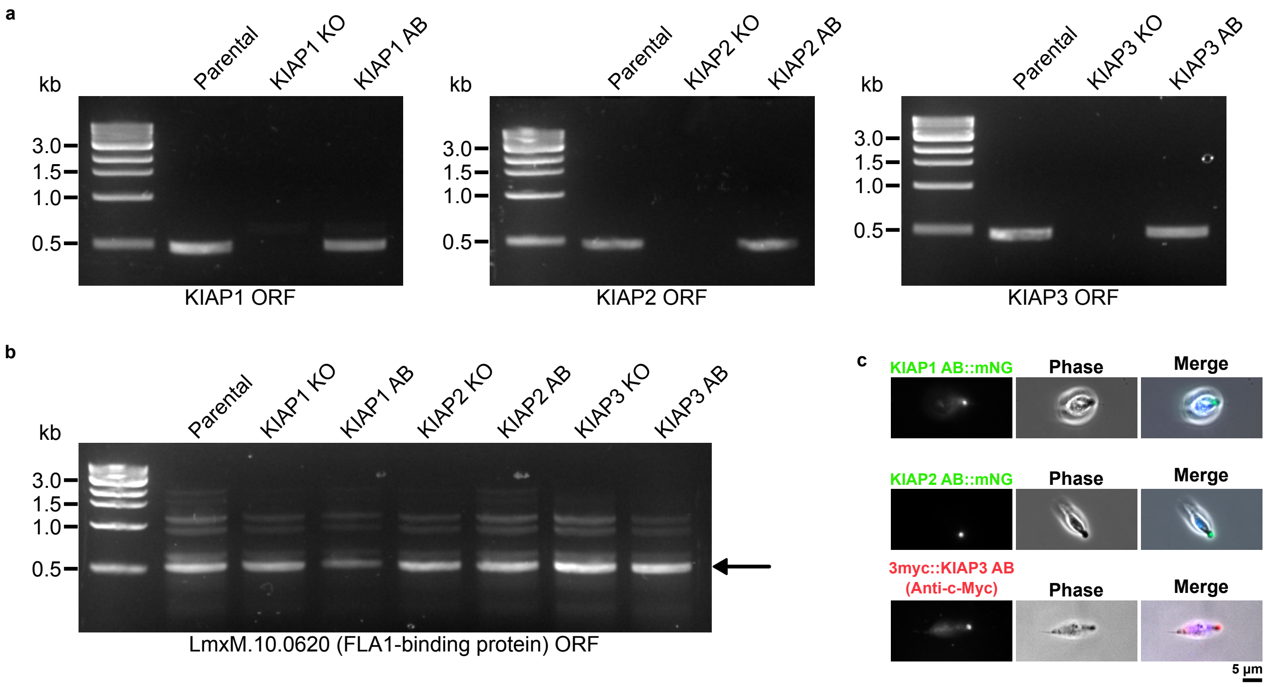

Supplementary Fig. 6. Confirmation of knockout and add back of KIAP1, 2 and 3 genes. **a**, Genomic DNA (gDNA) from parental and KIAP1, 2 and 3 knockout and add back cells was analysed by PCR. PCR confirmed that KIAP1, 2 and 3 open reading frame (ORF) was no longer present in knockout cells, and KIAP1, 2 and 3 ORF was present in add back cells. Source data are provided as a Source Data file. **b**, The presence of the ORF of FLA1-binding protein (LmxM.10.0620) was confirmed by PCR using the respective gDNA. The black arrow indicates the amplified 500 bp bands from the ORF of FLA1-binding protein. Source data are provided as a Source Data file. **c**, Fluorescence microscopy confirmed that the KIAP1::mNG::3myc, KIAP2::mNG::3myc and 3myc::KIAP3 expressed in add back cell localised to the attached flagellum of *in vitro* haptomonad-like promastigotes. Note that, to mitigate the reduced attachment capacity of *L. mexicana* cell line expressing KIAP3 N-terminally tagged with mNG and a triple myc tag, KIAP3 N-terminally tagged only with a triple myc tag was re-introduced in KIAP3 AB cells. Localisation of 3myc::KIAP3 in an *in vitro* haptomonad-like promastigote was confirmed by immunofluorescence microscopy using c-Myc monoclonal antibody (9E10) and Alexa Fluor 546-conjugated goat anti-mouse secondary antibody. Representative images from at least n = 2 independent sample preparations are shown. In the merged images, the overlays of the phase contrast, mNG (green) or Alexa Fluor 546 (red) and Hoechst-stained DNA (blue) images are shown.

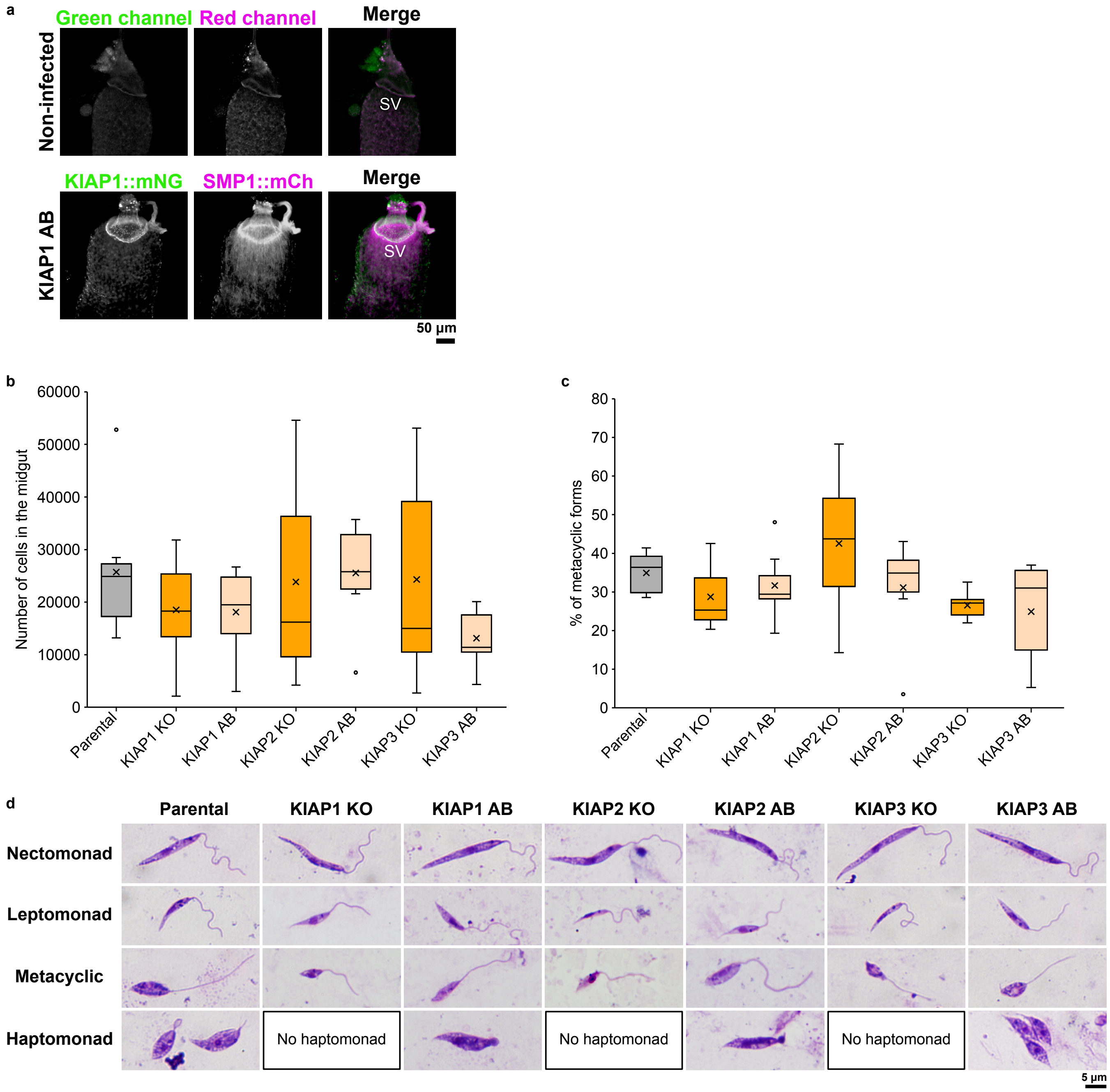

Supplementary Fig. 7. KIAP1::mNG on the stomodeal valve, metacyclic quantification, and morphological forms of *L. mexicana* occurring in *L. longipalpis* midguts during the late stage infection.. a, Fluorescence images of a sand fly midgut non-infected and infected with *L. mexicana* (KIAP1 AB) cells expressing KIAP1::mNG and SMP1::mCh on day 7 PBM. The location of the stomodeal valve is shown with SV. **b, c,** Quantification of the total number of parasites (b) and percentage of metacyclics in the midgut (c) on day 9 PBM. Boxes and whiskers indicate the median, upper and lower quartiles and 5th/95th percentiles. Crosses and dots indicate mean values and outliers, respectively. No significant differences in the number of cells in the midgut and the percentage of metacyclics were observed between the experimental groups (n = 7 infected midguts), P = 0.419 and 0.124, respectively, Kruskal-Wallis test. Source data are provided as a Source Data file. **d,** Nectomonad, leptomonad, and metacyclic promastigotes were observed in the midguts during the late stage infection in all cell lines (procyclic promastigotes were absent from our smears on day 9 PBM because they are present in sand flies only during the early stages of infection, prior to defecation). No haptomond promastigotes were found in any KIAP deletion cell lines. Representative images from n = 7 infected midguts in b.

Supplementary Reference

1. Nielsen, B. B. *et al.* Crystal structure of tetranectin, a trimeric plasminogen-binding protein with an α-helical coiled coil. *FEBS Lett* **412**, 388–396 (1997).

Source Data Supplementary Fig. 6. Uncropped scans of the gels.

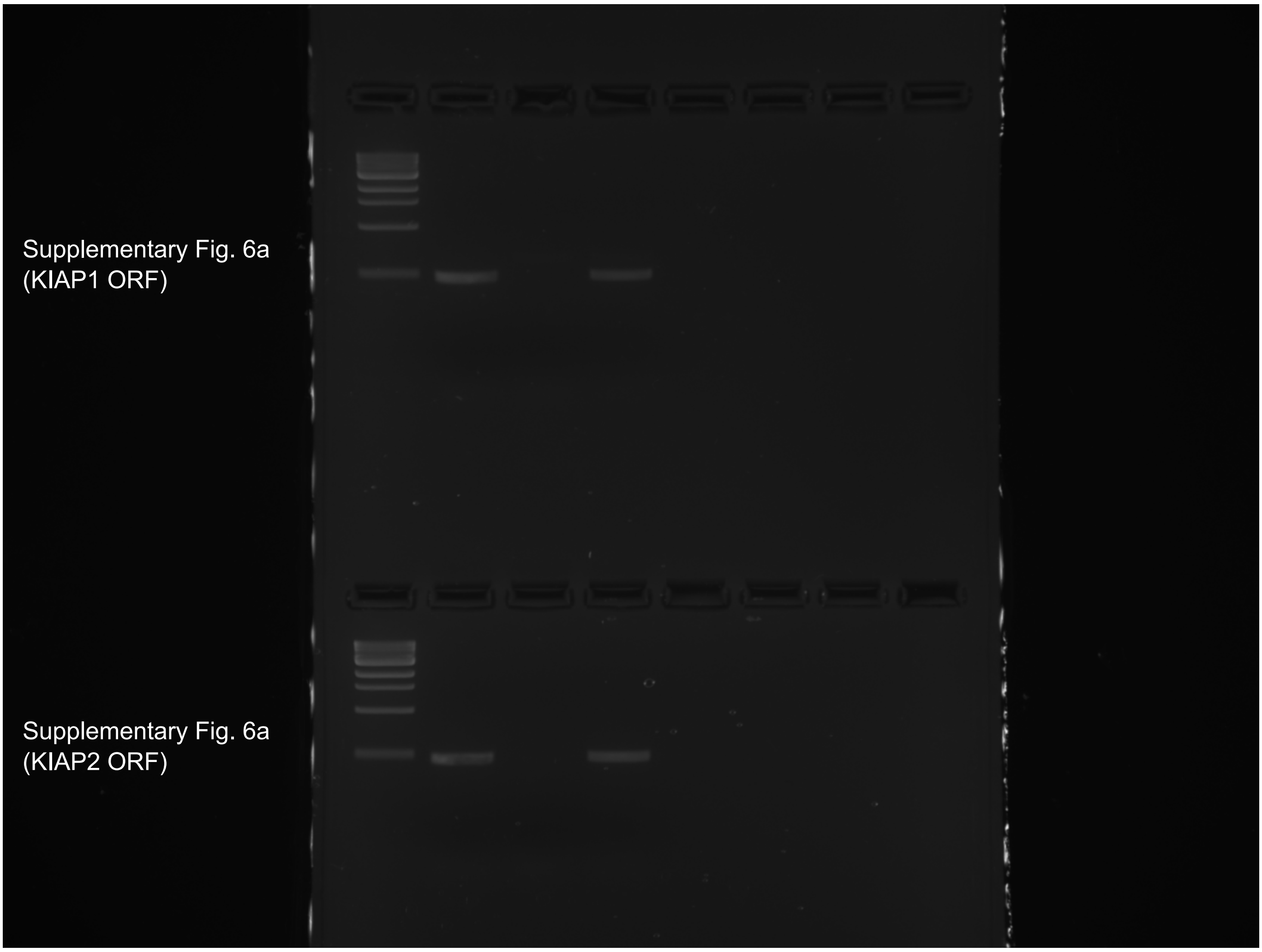

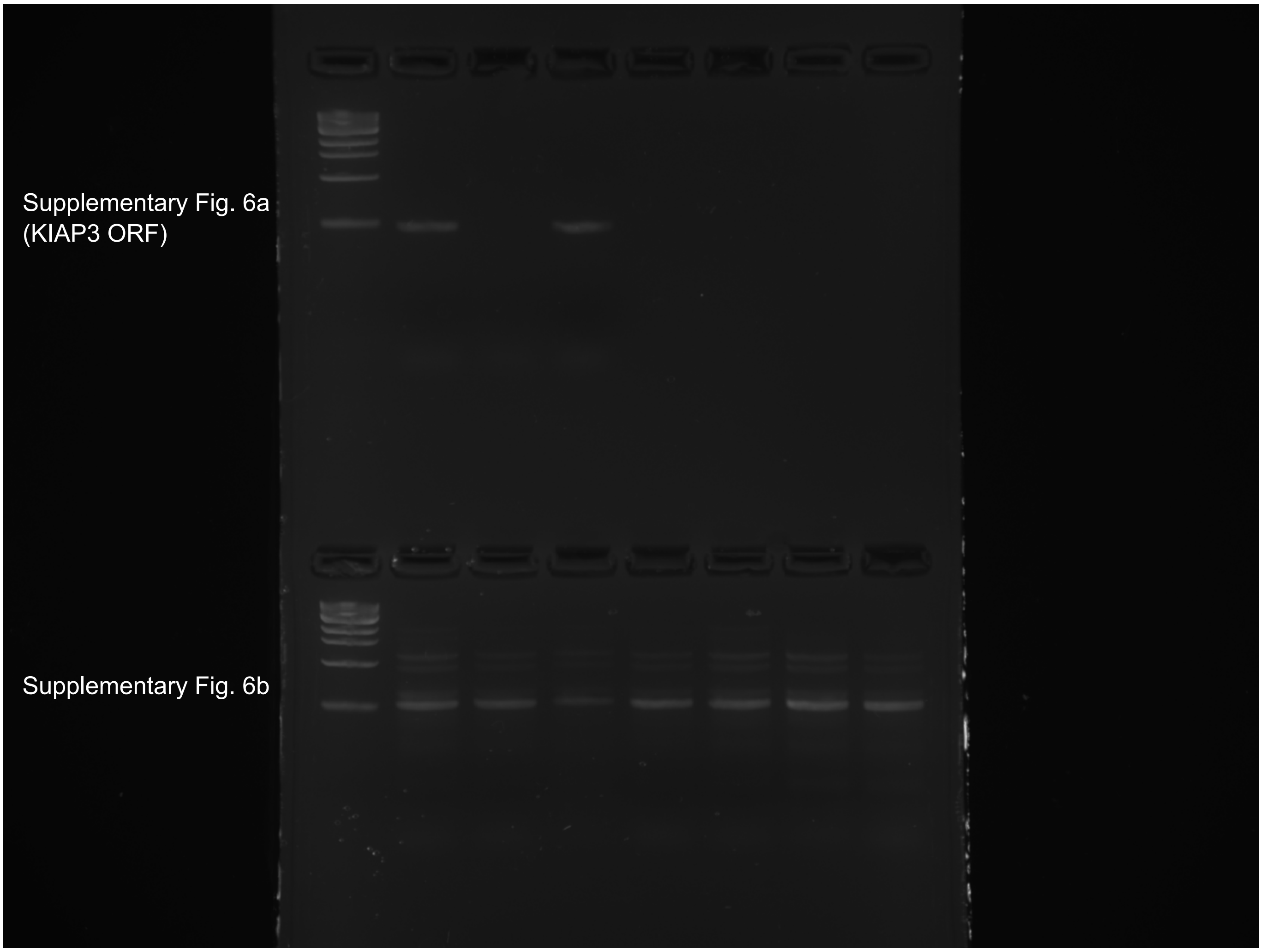

Supplement: Supplementary file 1 — Supplementary Information [file 41467_2024_51291_MOESM1_ESM.pdf]
