# Supplementary material for: Discovery of essential kinetoplastid-insect adhesion proteins and their function in Leishmania-sand fly interactions
Source: Nat Commun. 2024 Aug 13;15:6960. doi: 10.1038/s41467-024-51291-z (PMC11322530; doi:10.1038/s41467-024-51291-z)
Supplement: Supplementary file 3 — Description of Additional Supplementary Files [file 41467_2024_51291_MOESM3_ESM.docx]

**Description of Additional Supplementary Files**

**Supplementary Data 1. All proteins detected in the flagellum samples of attached *in vitro* haptomonad-like promastigotes and non-attached *in vitro* promastigotes.**

Proteins highlighted in gray or orange show the 371 proteins for which there are more peptide spectrum counts detected in the flagellum sample of attached *in vitro* haptomonad-like promastigotes (HA) versus the flagellum sample of non-attached *in vitro* promastigotes (PR), after removing the non-*Leishmania* proteins and regardless of the Fischer's exact test value. Proteins highlighted in orange shows the 20 proteins selected for mNG-tagging screening after applying a series of exclusion criteria shown in Supplementary Data 2 and 3.

**Supplementary Data 2. Proteins enriched by at least 8-fold in the attached flagellum sample versus the non-attached flagellum sample.**

Proteins highlighted in gray were excluded from further analysis. These include i) proteins with annotation or protein domain that suggest a function unrelated to attachment e.g. metabolism, mitochondrial, protein folding and assembly, endocytic trafficking, transcription, transporters, proteasome, cell cycle kinase; ii) proteins with a known function or localisation e.g. SMP1, intraflagellar transport (IFT) proteins, kinesins; iii) Pseudogenes and non-scaffolded genes. Proteins highlighted in orange were taken forward, with priority given to hypothetical proteins and those with potential cytoskeletal functions and cAMP signalling.

**Supplementary Data 3. 39 proteins taken forward after exclusion criteria in Supplementary Data 2 were applied.**

*Trypanosoma brucei* orthologs of these proteins were identified. The annotation and localisation of these orthologs was used to further refine the list. Proteins whose *T. brucei* ortholog localised to the mitochondrion, cytoplasm, endocytic system, or nucleus were excluded, as were orthologs annotated with functions unlikely to be related to attachment e.g. tRNA import. *Leishmania* specific proteins and those whose orthologs had a cytoskeletal localisation or background signal were prioritised. The excluded proteins are highlighted in gray, with those taken forward for tagging in orange.

**Supplementary Data 4. List of primers used for protein tagging, gene deletion, gene add back and deletion validation.**

**Supplementary Movie 1. Time-lapse movie of a complete adhesion process of an *in vitro* haptomonad-like promastigote expressing mNG::KIAP3.**

Playback of ~3 h at 500x speed.

**Supplementary Movie 2. Time-lapse movie of an initial adhesion process of an *in vitro* haptomonad-like promastigote expressing KIAP1::mNG and mCh::KIAP3.**

Playback of ~25 min at 100x speed.

**Supplementary Movie 3. Time-lapse movie of an initial adhesion process of an *in vitro* haptomonad-like promastigote expressing KIAP2::mNG.**

Playback of ~15 min at 100x speed.

**Supplementary Movie 4. Time-lapse movie of a complete adhesion process of an *in vitro* haptomonad-like promastigote expressing KIAP2::mNG.**

Playback of ~6 h at 1200x speed.

**Supplementary Movie 5. Movie of real-time observation of the parental *L. mexicana* infected thoracic midgut before and after midgut disruption on day 8 PBM.**

**Supplementary Movie 6. Movie of real-time observation of the KIAP1 KO *L. mexicana* infected thoracic midgut before and after midgut disruption on day 8 PBM.**

**Supplementary Movie 7. Movie of real-time observation of the KIAP1 AB *L. mexicana* infected thoracic midgut before and after midgut disruption on day 8 PBM.**

**Supplementary Movie 8. Movie of real-time observation of the KIAP2 KO *L. mexicana* infected thoracic midgut before midgut disruption on day 8 PBM.**

**Supplementary Movie 9. Movie of real-time observation of the KIAP2 AB *L. mexicana* infected thoracic midgut before midgut disruption on day 8 PBM.**

**Supplementary Movie 10. Movie of real-time observation of the KIAP3 KO *L. mexicana* infected thoracic midgut before and after midgut disruption on day 8 PBM.**

**Supplementary Movie 11. Movie of real-time observation of the KIAP3 AB *L. mexicana* infected thoracic midgut before and after midgut disruption on day 8 PBM.**
