# Supplementary material for: Discovery of essential kinetoplastid-insect adhesion proteins and their function in Leishmania-sand fly interactions
Source: Nat Commun. 2024 Aug 13;15:6960. doi: 10.1038/s41467-024-51291-z (PMC11322530; doi:10.1038/s41467-024-51291-z)
Supplement: Supplementary file 9 — Reporting Summary [file 41467_2024_51291_MOESM9_ESM.pdf]

nature portfolio

Corresponding author(s): Jack Sunter

Last updated by author(s): Jul 18, 2024

Reporting Summary

Nature Portfolio wishes to improve the reproducibility of the work that we publish. This form provides structure for consistency and transparency in reporting. For further information on Nature Portfolio policies, see our Editorial Policies and the Editorial Policy Checklist.

Statistics

For all statistical analyses, confirm that the following items are present in the figure legend, table legend, main text, or Methods section.

n/a	Confirmed	
☐	☒	The exact sample size (*n*) for each experimental group/condition, given as a discrete number and unit of measurement
☐	☒	A statement on whether measurements were taken from distinct samples or whether the same sample was measured repeatedly
☐	☒	The statistical test(s) used AND whether they are one- or two-sided<br>*Only common tests should be described solely by name; describe more complex techniques in the Methods section.*
☒	☐	A description of all covariates tested
☒	☐	A description of any assumptions or corrections, such as tests of normality and adjustment for multiple comparisons
☐	☒	A full description of the statistical parameters including central tendency (e.g. means) or other basic estimates (e.g. regression coefficient) AND variation (e.g. standard deviation) or associated estimates of uncertainty (e.g. confidence intervals)
☐	☒	For null hypothesis testing, the test statistic (e.g. F, t, r) with confidence intervals, effect sizes, degrees of freedom and P value noted<br>*Give P values as exact values whenever suitable.*
☒	☐	For Bayesian analysis, information on the choice of priors and Markov chain Monte Carlo settings
☒	☐	For hierarchical and complex designs, identification of the appropriate level for tests and full reporting of outcomes
☒	☐	Estimates of effect sizes (e.g. Cohen's d, Pearson's r), indicating how they were calculated

Our web collection on statistics for biologists contains articles on many of the points above.

Software and code

Policy information about availability of computer code

Data collection	Widefield microscopy images were captured using ZEN software (blue edition, Zeiss) or CellSens software (Olympus).<br>Confocal microscopy images were captured using ZEN software (black edition, Zeiss) or Leica Application Suite X Software (Leica).<br>Proteomics data acquisition was performed using Xcalibur software (version 4.0, Thermo Fisher Scientific).
Data analysis	Microscopy images were analysed using Fiji (1.54f).<br>Electron tomography data were analysed using 3dmod (IMOD software package, v4.11).<br>Tandem mass spectra were extracted using MSConvert to .mgf format before submitting to database searching using Mascot (Matrix Science, version 2.7.0.1).<br>Scaffold (version Scaffold_5.2.0, Proteome Software Inc.) was used to validate MS/MS based peptide and protein identifications.

For manuscripts utilizing custom algorithms or software that are central to the research but not yet described in published literature, software must be made available to editors and reviewers. We strongly encourage code deposition in a community repository (e.g. GitHub). See the Nature Portfolio guidelines for submitting code & software for further information.

Data

Policy information about availability of data

All manuscripts must include a data availability statement. This statement should provide the following information, where applicable:

- Accession codes, unique identifiers, or web links for publicly available datasets
- A description of any restrictions on data availability
- For clinical datasets or third party data, please ensure that the statement adheres to our policy

The mass spectrometry proteomics data sets generated in this study have been deposited in the MassIVE under accession code MSV000092919 [https://massive.ucsd.edu/ProteoSAFe/private-dataset.jsp?task=0e17d69a63e44c9b8cf0cc00ebe2ab37] and the ProteomeXchange Consortium under accession code PXD045552 [https://proteomecentral.proteomexchange.org/cgi/GetDataset?ID=PXD045552]. The tomography data sets generated in our previous study have been deposited in the EMPIAR under accession code EMPIAR-11467 [https://www.ebi.ac.uk/empiar/EMPIAR-11467/] and EMPIAR-11468 [https://www.ebi.ac.uk/empiar/EMPIAR-11468/]. Source data are provided with this paper.

Research involving human participants, their data, or biological material

Policy information about studies with human participants or human data. See also policy information about sex, gender (identity/presentation), and sexual orientation and race, ethnicity and racism.

Reporting on sex and gender	This research does not involve human participants, their data, or biological material.
Reporting on race, ethnicity, or other socially relevant groupings	N/A
Population characteristics	N/A
Recruitment	N/A
Ethics oversight	N/A

Note that full information on the approval of the study protocol must also be provided in the manuscript.

Field-specific reporting

Please select the one below that is the best fit for your research. If you are not sure, read the appropriate sections before making your selection.

[x] Life sciences [ ] Behavioural & social sciences [ ] Ecological, evolutionary & environmental sciences

For a reference copy of the document with all sections, see nature.com/documents/nr-reporting-summary-flat.pdf

Life sciences study design

All studies must disclose on these points even when the disclosure is negative.

Sample size	Sample sizes was determined by reference to previously published work in this field and were of a similar size. Two to four independent experiments were combined depending on analysis.
Data exclusions	No data were excluded.
Replication	All experiments were performed at least two times unless otherwise specified in the figure legend. All attempts at replication were successful.
Randomization	For widefield microscopy data collection random fields of view were captured. Randomisation/participant allocation was not performed as it was not relevant to this study. Controls, replicates and statistical methods ensured the validity of our results while minimizing the effect of variables.
Blinding	No blinding occurred in this study. Given the replication of experiments, objectivity of our measurements, standardised protocols, and data analysis, blinding was not considered necessary as it would not provide significant benefits in minimising bias.

Reporting for specific materials, systems and methods

We require information from authors about some types of materials, experimental systems and methods used in many studies. Here, indicate whether each material, system or method listed is relevant to your study. If you are not sure if a list item applies to your research, read the appropriate section before selecting a response.

Materials & experimental systems

n/a	Involved in the study	
☐	☒	Antibodies
☐	☒	Eukaryotic cell lines
☒	☐	Palaeontology and archaeology
☐	☒	Animals and other organisms
☒	☐	Clinical data
☒	☐	Dual use research of concern
☒	☐	Plants

Methods

n/a	Involved in the study	
☒	☐	ChIP-seq
☒	☐	Flow cytometry
☒	☐	MRI-based neuroimaging

Antibodies

Antibodies used	c-Myc Monoclonal Antibody (9E10) (Invitrogen, MA1-980)<br>Alexa Fluor 546-conjugated goat anti-mouse secondary antibody (Invitrogen, A-11030)
Validation	Antibodies have been validated by the supplier (https://www.thermofisher.com/order/genome-database/dataSheetPdf?producttype=antibody&productsubtype=antibody_primary&productId=MA1-980&version=Local).

Eukaryotic cell lines

Policy information about cell lines and Sex and Gender in Research

Cell line source(s)	Leishmania mexicana (WHO strain MNYC/BZ/1962/M379, expressing Cas9 and T7 RNA polymerase)<br>Leishmania major (pTB007 cell line, expressing Cas9 and T7 RNA polymerase)
Authentication	Genome and mRNA sequencing prior to the study
Mycoplasma contamination	Cell lines were monitored for contamination, including mycoplasma contamination, through DNA staining and microscopy during data capture.
Commonly misidentified lines (See ICLAC register)	N/A

Animals and other research organisms

Policy information about studies involving animals; ARRIVE guidelines recommended for reporting animal research, and Sex and Gender in Research

Laboratory animals	Insects - Female Lutzomyia longipalpis (Jadobina strain (between 3 and 5 days old)).
Wild animals	The study does not use animals collected from the wild.
Reporting on sex	Only female sand flies were used in the infection experiments.
Field-collected samples	The study does not use animals collected from the field.
Ethics oversight	No ethical approval was required for the use of sand flies.

Note that full information on the approval of the study protocol must also be provided in the manuscript.
